# A systematic review of structural and functional magnetic resonance imaging studies on the neurobiology of depressive symptoms in schizophrenia spectrum disorders

**DOI:** 10.1038/s41537-024-00478-w

**Published:** 2024-07-04

**Authors:** Julia Gallucci, Maria T. Secara, Oliver Chen, Lindsay D. Oliver, Brett D. M. Jones, Tulip Marawi, George Foussias, Aristotle N. Voineskos, Colin Hawco

**Affiliations:** 1 Campbell Family Mental Health Research Institute, Toronto, ON Canada; 2 Institute of Medical Science, University of Toronto, Toronto, ON Canada; 3 Department of Psychiatry, University of Toronto, Toronto, ON Canada

**Keywords:** Schizophrenia, Biomarkers

## Abstract

Depressive symptoms in Schizophrenia Spectrum Disorders (SSDs) negatively impact suicidality, prognosis, and quality of life. Despite this, efficacious treatments are limited, largely because the neural mechanisms underlying depressive symptoms in SSDs remain poorly understood. We conducted a systematic review to provide an overview of studies that investigated the neural correlates of depressive symptoms in SSDs using neuroimaging techniques. We searched MEDLINE, PsycINFO, EMBASE, Web of Science, and Cochrane Library databases from inception through June 19, 2023. Specifically, we focused on structural and functional magnetic resonance imaging (MRI), encompassing: (1) T1-weighted imaging measuring brain morphology; (2) diffusion-weighted imaging assessing white matter integrity; or (3) T2*-weighted imaging measures of brain function. Our search yielded 33 articles; 14 structural MRI studies, 18 functional (f)MRI studies, and 1 multimodal fMRI/MRI study. Reviewed studies indicate potential commonalities in the neurobiology of depressive symptoms between SSDs and major depressive disorders, particularly in subcortical and frontal brain regions, though confidence in this interpretation is limited. The review underscores a notable knowledge gap in our understanding of the neurobiology of depression in SSDs, marked by inconsistent approaches and few studies examining imaging metrics of depressive symptoms. Inconsistencies across studies’ findings emphasize the necessity for more direct and comprehensive research focusing on the neurobiology of depression in SSDs. Future studies should go beyond “total score” depression metrics and adopt more nuanced assessment approaches considering distinct subdomains. This could reveal unique neurobiological profiles and inform investigations of targeted treatments for depression in SSDs.

## Introduction

Depressive symptoms are highly prevalent in individuals with schizophrenia spectrum disorders (SSDs)^1^, with as many as 80% of patients experiencing a depressive episode at some point during their course of illness^2,3^. Depression and depressive symptoms in SSDs are associated with poorer outcomes^4^, including reduced quality of life^5,6^, increased burden of disease^1^, and a higher frequency of both self-harm^7,8^ and suicide^9,10^. Yet, our understanding and diagnosis of depression and depressive symptoms in individuals with SSDs are limited, with therapeutic options providing little efficacy^11–13^.

Diagnosing and treating depression in SSDs has posed a challenge^14^. This complexity entails not only identifying general depressive symptoms but also distinguishing them from comorbid depressive disorders^15^ as well as core symptom dimensions of schizophrenia, namely negative symptoms^16–18^. While antidepressant medications, the mainstay approach for treating major depressive disorders (MDD), are often prescribed for depression in SSDs^19^, findings from recent reviews revealed minimal to modest clinical improvements^11,12^. Importantly, findings from the Recovery After an Initial Schizophrenia Episode (RAISE) trial, an early treatment program for first-episode psychosis, suggested that less frequent antidepressant use may be linked to fewer side effects^13^.

Neuroimaging methods could enhance our comprehension of the pathophysiological mechanisms linked to depression in SSDs^20^. For instance, in MDD, identifying neuroimaging correlates of antidepressant treatment responses has enabled researchers to gain insights into how antidepressants impact select brain regions, perhaps leading to improved symptom outcomes^21^. Moreover, neuroimaging can serve as a tool to guide nonpharmacological interventions, such as repetitive transcranial magnetic stimulation (rTMS), allowing for more precise and individualized targeting of symptom-related circuits that optimize treatment response^22–25^. In light of robust evidence that rTMS mitigate depressive symptoms in MDD^26^ and preliminary support in SSDs^27^, further investigation into neuroimaging correlates may inform the selection of neurostimulation targets. While our knowledge regarding the neural mechanisms underlying depression in SSDs is limited^28^, gaining a deeper understanding has the potential to enhance opportunities for effective intervention^4^.

To our knowledge, there has not been a comprehensive synthesis of existing literature on the neurobiological underpinnings of depressive symptoms in SSDs. Therefore, we conducted a systematic review to provide an overview of studies that investigate the neural correlates of depressive symptoms in SSDs using neuroimaging techniques. Specifically, we focused on structural and functional magnetic resonance imaging (MRI), encompassing T1-weighted imaging studies evaluating brain morphology (e.g., volume or thickness), diffusion MRI (dMRI) studies examining white matter metrics (e.g., fractional anisotropy (FA) or mean diffusivity (MD)), and T2*-weighted imaging studies assessing brain function (e.g., activity or connectivity).

## Methods

### Registration

This systematic review was conducted per the guidelines outlined in the Preferred Reporting Items for Systematic Reviews and Meta-Analyses (PRISMA)^29^ and registered on the International Prospective Register of Systematic Reviews (PROSPERO: CRD42023433464). Before registration and screening, a librarian from the Center for Addiction and Mental Health (CAMH) in Toronto, Canada reviewed the search strategy and protocol.

### Information sources and search strategy

**Fig. 1:**
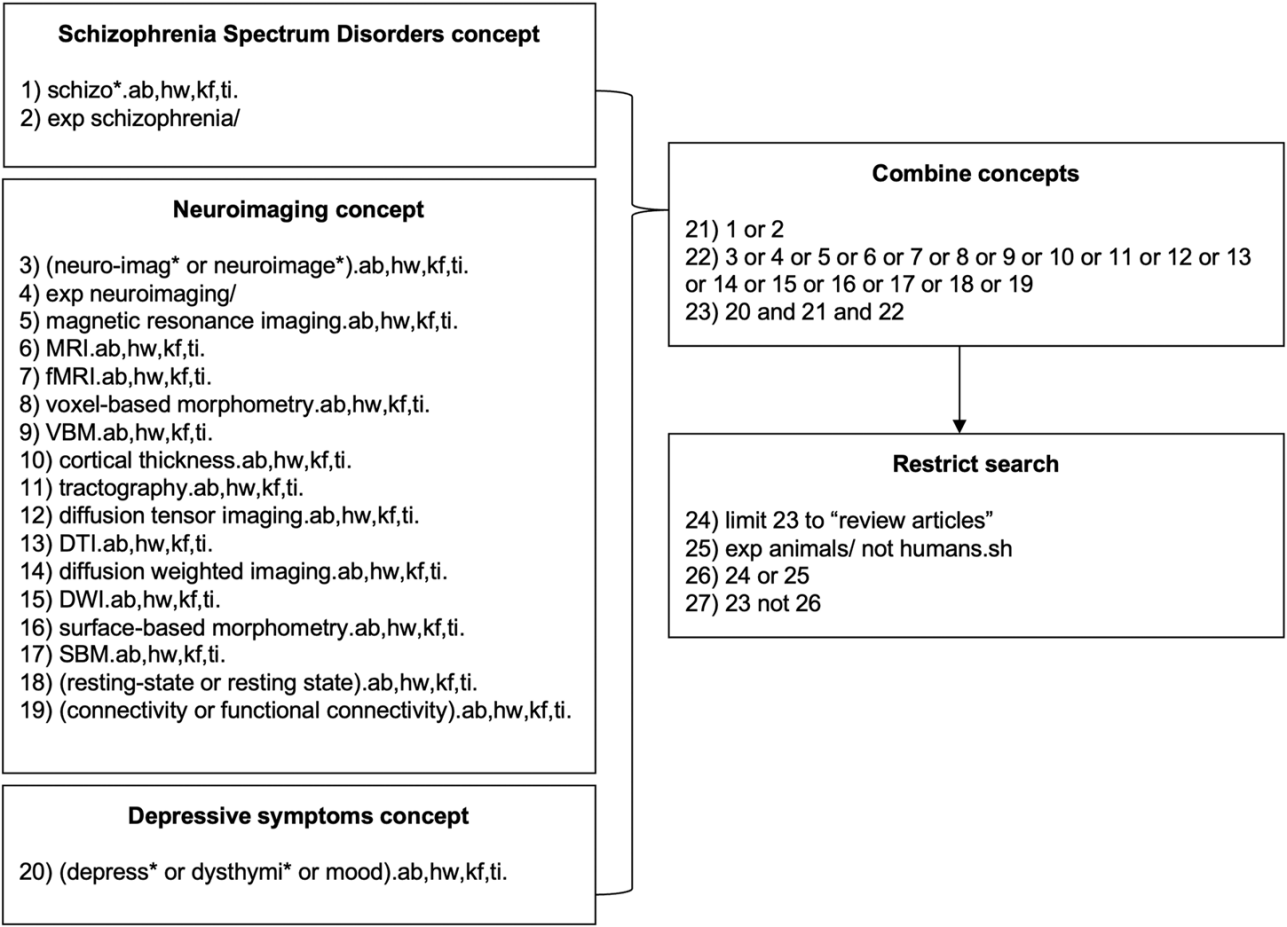
Search concepts. Medical Subject Headings (MeSH) and key terms adapted for MEDLINE (Ovid). Ab indicates abstract; hw, subject heading word; kf, keyword heading word; ti, title.

A systematic review of the literature was conducted using MEDLINE (Ovid), PsycINFO (Ovid), EMBASE (Ovid), Web of Science, and Cochrane Library electronic databases from inception through June 19, 2023. Figure 1 outlines the detailed search strategy used for MEDLINE; additional strategies tailored to other databases can be found in the Supplemental Materials. In summary, our search strategy encompassed Medical Subject Headings (MeSH) and keywords related to three main search blocks: SSDs, neuroimaging (structural and functional MRI, methodology of interest), and depressive symptoms (primary outcome measure). Additionally, we conducted backward and forward citation searches for all eligible studies that met the inclusion criteria.

### Eligibility criteria and study selection

Studies were included if they met the following criteria: (1) all participants were adults aged 18 years or older; (2) inclusion of a group with SSDs (i.e., schizophrenia, schizoaffective disorder, schizophreniform disorder, delusional disorder, brief psychotic disorder, schizotypal or schizoid personality disorder, or psychosis not otherwise specified) or first episode psychosis based on criteria from the Diagnostic and Statistical Manual of Mental Disorders (DSM); (3) study assessed depressive symptoms using a clinical rating measure (e.g., Calgary Depression Scale for Schizophrenia (CDSS) or Hamilton Depression Rating Scale (HAMD)); (4) study utilized one (or more) of the following structural/functional MRI modalities: T1-weighted scans measuring brain morphology (e.g., volume or thickness), diffusion-weighted scans measuring white matter integrity (e.g., FA or MD), or T2*-weighted scans measuring brain function (e.g., activity or connectivity); and (5) study reported findings from an analysis investigating the association between imaging measures and depressive symptoms.

Studies were excluded if they: (1) included participants diagnosed with a major neurological illness (e.g., stroke, Parkinson’s disease, epilepsy, multiple sclerosis, traumatic brain injury); (2) reported on case studies or non-human subjects. Conference abstracts, commentaries, opinion pieces, letters to the editor, and reviews were also excluded. As we conducted the review, we added an additional exclusion criterion to exclude transdiagnostic studies in which SSDs did not constitute at least 75% of the sample, and the effects in SSDs were not reported separately (i.e., the association between an imaging measure and depressive symptoms was observed across multiple diagnoses and did not specify SSDs).

### Data selection

Following the removal of duplicate entries, studies identified through electronic database searches underwent initial screening based on their titles and abstracts. This screening was carried out independently by two reviewers (JG and OC), who assessed the studies for their relevance with regard to the study population, condition, methodology, and outcomes of interest. Any discrepancies between assessments were resolved by a third reviewer (LDO). Subsequently, a full-text review of studies included from the initial screening stage was conducted independently by two reviewers (JG and MTS). In cases where uncertainty regarding eligibility arose, a third reviewer (TM) resolved the discrepancies. A covidence reference management system was used throughout the screening and selection process of the studies.

### Data extraction

Data from studies that satisfied the inclusion criteria were extracted and recorded in a database by 1 of 2 reviewers (JG and OC) and subsequently cross-checked by the other. This database encompassed details including bibliographic information, study type, sample size, mean age of the groups, sex distribution, medication usage details, diagnostic criteria or assessment tools for SSDs evaluation, scales or assessments for measuring depressive symptoms, imaging modality, imaging analysis and processing approach, statistical analysis methods, and a summary of study findings.

### Quality assessment

For assessing the quality and risk of bias in the included articles, a modified version of the Newcastle Ottawa Scale (NOS) for cohort studies was utilized, performed by either JG or OC (see Supplemental Table S1 for details). The questions regarding the ‘non-exposed’ cohort were removed, as we were only interested in SSD-specific findings. Additionally, the scale was modified to check the adequacy of the sample size per group, and a point was given to studies with a sample size of 30 or greater^30,31^. Since medication usage is an important confounding factor in the link between depressive symptom severity and brain metrics^32,33^, one question was added to assess whether medication information was acquired in the studies. A point was also given to studies that had used a validated clinical scale when assessing depressive symptoms. The modified NOS score ranged from 1 to 8, indicating low to high quality. In summary, points were allocated to each study and summed up to range from 0–8, with scores between 0-3 indicating poor quality; 4–5, moderate quality; and 6+, good quality. Any uncertainties that arose during the assessment were discussed between the two reviewers (JG and OC) until a consensus was reached.

## Results

### Overview of study characteristics

**Fig. 2:**
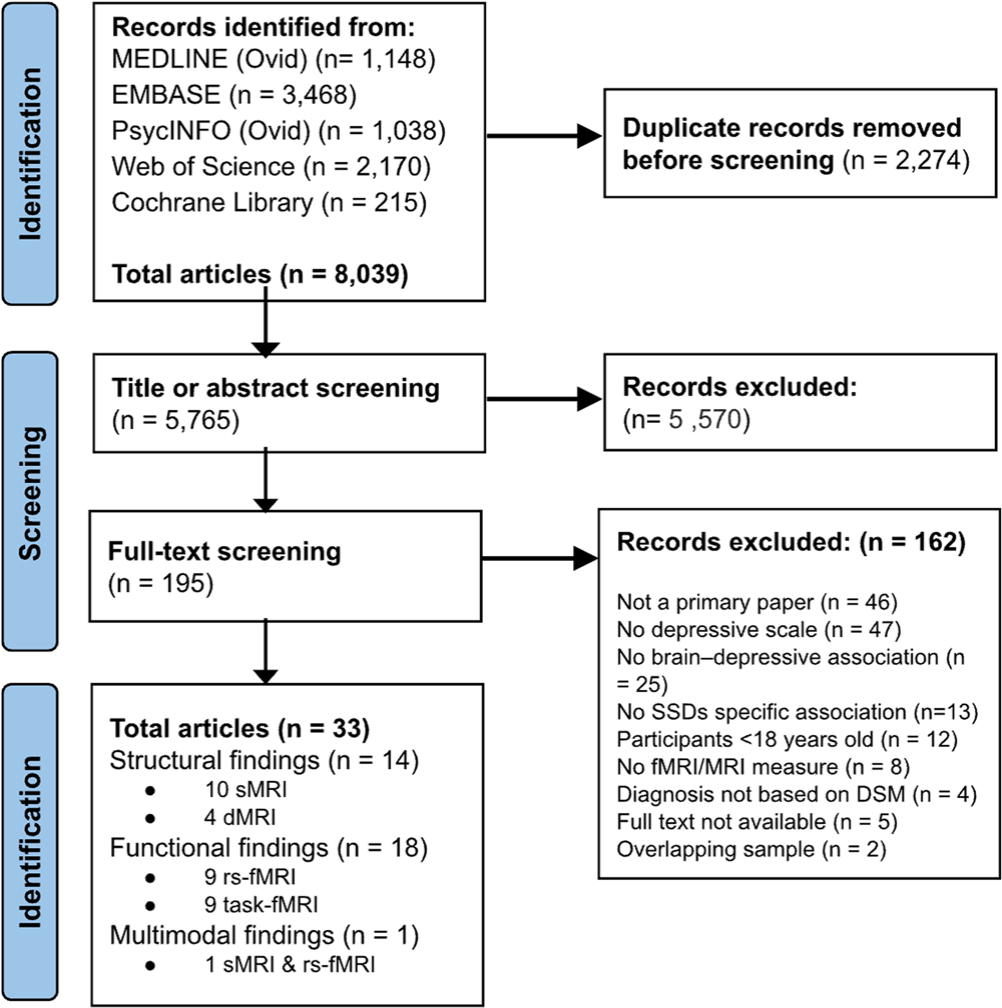
PRISMA flowchart illustrating the inclusion and exclusion of records through different screening stages, leading to a final compilation of articles for inclusion and subsequent data extraction. DSM Diagnostic and Statistical Manual of Mental Disorders, dMRI diffusion MRI, fMRI functional magnetic resonance imaging, rs-fMRI resting-state fMRI, sMRI structural MRI, SSDs schizophrenia spectrum disorders.

Our initial search identified 5,765 potentially relevant studies, excluding duplicates. Following a review of titles and abstracts, 5570 studies were excluded, leaving 195 for full-text screening. After this stage, 162 studies were excluded, resulting in a final selection of 33 studies. Among these, 14 studies used T1-weighted structural MRI (sMRI) or dMRI, 18 used fMRI, and one study used both fMRI and sMRI (Fig. 2). Tables 1 and 2 present the characteristics of the structural and functional neuroimaging studies, respectively, including the number of participants, sex distribution, mean age, and quality assessment scores. Across included studies, sample sizes tended to be small (a group of less than 30 participants in 15 out of 33 studies), yet exhibited a wide variation (overall sample sizes ranged from 15 to 312 participants). Most study participants were male (1293 of 2007; 64%) and had an average age of 33.7 土 11.2. Antipsychotic medication use was reported in 21 studies, with 19 using chlorpromazine, one using olanzapine, and one using haloperidol equivalents; two studies specified that participants were drug or neuroleptic naive. Additionally, six studies reported the use of antidepressant medication. All studies used either DSM-III, DSM-IV, or DSM-5 for SSDs diagnosis (details provided in Tables 1–2). Thirty-one studies recruited patients with schizophrenia. Six studies included patients with schizoaffective disorder and one with schizophreniform disorder. Four studies designated patients as first-episode schizophrenia or psychosis. None of the studies included individuals with comorbid depressive disorders.

**Table 1:** Neural correlates of depressive symptoms in 15 studies with structural MRI.

Source	No. of SSDs	Diagnostic Tool	Male, No./Total No. (%)	Age, mean (SD), y	QA score^†^	Main depressive symptoms measure(s)	Main neuroimaging measure(s)	Main finding
*sMRI*								
Buck et al., 2022^a ^^35^	100 FEP Non-affective 80 Affective 20	DSM-IV	75/100 (75.0)	Male 23.9 (3.9) Female 24.5 (4.2)	5	CDSS	Whole-brain cortical and hippocampal subfield volume and thickness	Female-specific FEP pattern of associations between fewer depressive symptoms coupled with reduced hippocampal subfields and both high and low thickness in specific cortical regions. The pattern of hippocampal and cortical anatomy included reduced volumes of the bilateral mammillary bodies, right fornix, and CA2/3 and left alveus, low thickness in the right superior temporal gyrus, entorhinal cortex, pars orbitalis, medial orbitofrontal gyrus and cingulate cortex, and high thickness in the left precentral gyrus, paracentral gyrus, cuneus, and lingual gyrus.
Voineskos et al., 2021^b^ ^27^	83 SCZ & SAD Active 41 Sham 40	DSM-IV	Active 30/41 (73.2) Sham 28/40 (70.0)	Active 37.1 (10.3) Sham 38.0 (11.2)	7	CDSS	Regional thickness of the dorsolateral prefrontal cortex	An increase in the right dorsolateral prefrontal cortex thickness was significantly associated with a reduction in CDSS total score in the active repetitive transcranial magnetic stimulation group.
Wei et al., 2021^c^ ^65^	42 FES Dep– 31 Dep+ 11	DSM-IV	Dep– 8/31 (25.8) Dep+ 11/11 (100.0)	Dep– 29.3 (10.3) Dep+ 26.6 (10.0)	7	HAMD	Whole-brain volume-based and surface-based morphometry	Drug naive FES patients with comorbid depressive symptoms had higher gray matter volume in the left isthmus cingulate and the left posterior cingulate cortex compared with drug naive FES patients without comorbid depressive symptoms. Drug naive FES patients with comorbid depressive symptoms showed the greater surface area in the left isthmus cingulate cortex, the left superior parietal gyrus, and the right cuneus than drug naive FES patients without comorbid depressive symptoms. Surface area and gray matter volume of the left isthmus cingulate were significantly correlated with the total score of HAMD in drug naive FES patients with comorbid depressive symptoms.
Siddi et al., 2019^64^	24 SCZ	DSM-IV	14/24 (58.3)	41.1 (9.7)	6	CDSS	Whole-brain gray matter and white matter volume	Depression score was inversely associated with the right medial superior frontal and right orbitofrontal gyrus volume, and, at a trend level, with the right middle frontal and superior frontal gyrus volume.
Bossu et al., 2015	71 SCZ	DSM-IV	45/71 (63.4)	41.9 (11.6)	6	PANSS- depression factor score	Regional volume of the hippocampus	Patients with a lower depression factor score showed an increased volume of both the left and right hippocampus.
Tomasino et al., 2011^62^	69 SCZ	DSM-IV	46/69 (66.7)	40.5 (12.1)	7	BPRS-depression and anxiety subscale	Regional volume of the amygdala	BPRS scores for depression–anxiety were significantly positively correlated with both right and left amygdalar volumes in SCZ.
Smith et al., 2003^36^	33 FES	DSM-IV	26/33 (78.8)	22.8 (4.9)	5	PANSS- depression and anxiety subscale	Regional size of hippocampal fissure	Significant association between increased hippocampal fissure size and more anxiety–depression in FES patients.
Ichimiya et al., 2001^37^	20 SCZ & SCZ-F	DSM-IV	20/20 (100.0)	28.3 (6.9)	4	BPRS-depression and anxiety subscale	Regional volume of the cerebellum	Significant negative correlation was found between the volume of the vermis and depression subscale in male neuroleptic-naive SCZ/SCZ-F patients.
Gur et al., 2000^61^	70 SCZ	DSM-IV	40/70 (57.1)	28.7 (6.9)	8	HAMD	Regional volume within sectors of the prefrontal lobe	Lower orbitofrontal volume was associated with a more depressed mood in patients.
Kohler et al.,1998^d^ ^60^	79 SCZ Dep– 39 Dep+ 40	DSM- III/IV	Dep– 26/39 (66.7) Dep+ 24/40 (60.0)	Dep– 30.0 (6.2) Dep+ 29.9 (7.4)	6	HAMD	Whole-brain volume	The SCZ high depression group had more temporal lobe volume on the left, and a trend for increased right temporal lobe volume compared to the SCZ low depression group.
*dMRI*								
Joo et al., 2021^59^	47 SCZ	DSM-5	35/47 (74.5)	36.0 (9.0)	6	BPRS-depression and anxiety subscale	Whole-brain tractography	In the SCZ group, there were associations of the fractional anisotropy and radial diffusivity of the left external capsule with the anxiety-depression score of the BPRS, which did not survive the correction for multiple tests.
Amodio et al., 2018^38^	30 SCZ	DSM-IV	30/30 (100.0)	37.0 (7.9)	4	PANSS- depression score	White matter connectivity patterns within the ventral tegmental area, striatum, orbitofrontal cortex, amygdala, and insular cortex	No correlation was found between PANSS depression scores, and any of the connectivity values obtained (lateral and medial orbitofrontal cortex, dorsal-lateral prefrontal cortex, along with ventral-anterior, dorsal-anterior, and posterior insular cortex regions of interest) in patients with chronic SCZ.
Long et al., 2018^e ^^39^	63 FES SI– 45 SI + 18	DSM-IV	SI– 25/45 (55.6) SI + 9/18 (50.0)	SI– 24.6 (5.9) SI + 24.8 (6.9)	5	CDSS	Whole-brain white matter fractional anisotropy and mean diffusivity	Compared with FES patients without suicidal ideation, the FES patients with suicidal ideation showed higher fractional anisotropy in the corpus callosum (genu, body, and splenium), left anterior corona radiata, left superior corona radiata and bilateral posterior corona radiata as well as lower mean diffusivity in the splenium of the corpus callosum, bilateral posterior corona radiata, left posterior thalamic radiation and left superior longitudinal fasciculus.
Chiappelli et al., 2014^58^	126 SCZ	DSM version not specified	70/126 (55.5)	37.9 (13.3)	6	MTSD	Whole-brain white matter fractional anisotropy	Greater trait depression was significantly and positively associated with the whole-brain average fractional anisotropy values and fractional anisotropy values for four white matter tracts— the corona radiata, thalamic radiation, superior longitudinal fasciculus, and superior frontal-occipital tract.
*sMRI & rs-fMRI*								
Liang et al., 2023^f ^^49^	312 SCZ & SAD SCZ 178 SAD 134	DSM-IV	SCZ 124/178 (69.7) SAD 58 /134 (37.7)	SCZ 34.5 (12.0) SAD 36.3 (12.3)	5	MADRS	Whole-brain gray matter volume	In SCZ, MADRS scores were positively associated with positive gray matter volume in the insula and inferior frontal cortex. In SAD, MADRS scores were positively associated with positive gray matter volume in the lingual and frontal gyrus.

^†^ the maximum QA score was 8 points, with lower scores reflecting a greater risk of bias.

^a^study split SSDs sample by sex.

^b^study split SSDs sample by treatment group (active or sham rTMS).

^c^study split SSDs sample by HAMD cut off of 20 into depressive (Dep +) or no depressive (Dep –) symptoms.

^d^study split SSDs sample by HAMD cut off of 18 into High depressive (Dep +) or Low depressive (Dep –) symptoms.

^e^study split SSDs sample by CDSS suicide item score cut off of 0 into suicidal ideation (SI +) or no suicidal ideation (SI –).

^f^study is multimodal; shown across both tables to demonstrate sMRI (bolded) and rs-fMRI results separately. Results reported for schizophrenia (SCZ) and schizoaffective disorder (SAD) separately

gray rows indicate studies with null findings.

*FEP* First Episode Psychosis, *FES* First Episode Schizophrenia, *SAD* Schizoaffective Disorder, *SCZ* Schizophrenia, *SCZ-F* Schizophreniform Disorder, *BPRS* Brief Psychiatric Rating Scale, *CDSS* Calgary Depression Scale for Schizophrenia, *HAMD* Hamilton Depression Rating Scale, *MADRS* Montgomery-Asberg Depression Rating Scale, *MTSD* Maryland Trait and State Depression Scale, *PANSS* Positive and Negative Symptom Scale.

**Table 2:** Neural correlates of depressive symptoms in 19 studies with functional MRI.

Source	No. of SSDs	Diagnostic Tool	Male, No./Total No. (%)	Age, mean (SD), y	QA score^†^	Main depressive symptoms measure(s)	Main neuroimaging measure(s)	Main finding
*rs-fMRI*								
Li et al., 2023^57^	88 SCZ	DSM-IV	38/88 (43.2)	28.8 (8.3)	6	PANSS- depression factor score	Whole-brain amplitude of low-frequency fluctuations (ALFF)	The degree of ALFF increase in the dorsolateral region of the superior frontal gyrus after treatment with second-generation antipsychotics was significantly and positively related to the magnitude of the decrease in depression factor score.
Doucet et al., 2020^56^	76 SCZ	DSM-5	57/76 (75.0)	26.9 (7.0)	7	BPRS-depression and anxiety subscale	Network-based functional connectivity	Strong mode of covariation linking functional network connectivity to clinical response; and particularly to improvement in positive and anxious/depressive symptoms. Higher internal cohesiveness of the default mode network was the single most important positive predictor. Key negative predictors involved the functional cohesiveness of central executive subnetworks anchored in the frontoparietal cortices and subcortical regions (including the thalamus and striatum) and the inter-network integration between the default mode and sensorimotor networks.
Lee et al., 2019^a ^^55^	79 SCZ Good 25 Mod 31 Poor 23	DSM-IV	Good 15/25 (60.0) Mod 16/31 (51.6) Poor 12/23 (52.2)	Good 41.0 (7.1) Mod 37.8 (7.5) Poor 37.3 (7.0)	6	BPRS- affect subscale	Functional connectivity of the default mode network	Default mode network connectivity in the right ventromedial prefrontal cortex negatively correlated with affect subscale scores in SCZ.
Xu et al., 2019^34^	84 SCZ & SAD	DSM-IV	59/84 (70.2)	34.9 (11.0)	3	PANSS- depression factor score	Seed-based functional connectivity of substantia nigra/ventral tegmental area	No correlation was found between depressive symptoms and substantia nigra/ventral tegmental area-seeded resting state functional connectivity.
Lee et al., 2018^54^	85 SCZ	DSM-5	64/85 (75.3)	27.2 (7.3)	7	BPRS-depression and anxiety subscale	Network-based functional connectivity	The best stepwise multiple regression model explained 12% of the variance in depressive symptom severity in patients with recent-onset schizophrenia. The neuroimaging variables that made a statistically significant contribution to the model were the within-network connectivity of the salience network and the connectivity between salience-language networks and somatomotor-auditory networks. For any unit increase in any of these connectivity measures, the model predicted lower scores in BPRS anxiety/depression symptoms.
Ohta et al., 2018^40^	21 SCZ	DSM-IV	11/21 (52.4)	42.3 (10.2)	5	CDSS	Functional connectivity of the salience network	Structural equation model revealed significant and moderate-to-strong negative associations between the right pallidum functional connectivity and CDSS total scores, and between CDSS total scores and quality of life scale scores.
Son et al.,2017^53^	41 SCZ	DSM-IV	20/41 (48.8)	37.5 (8.6)	7	PANSS- depression factor score	Functional connectivity of the frontoparietal network	Mean connectivity of the left frontoparietal network was negatively correlated with the score of PANSS depressive factor in SCZ.
Su et al., 2015^41^	49 SCZ	DSM-IV	22/49 (44.9)	38.4 (13.4)	5	PANSS- depression and anxiety subscale	Whole-brain Small-world network metrics (global and network efficiency)	Depression and anxiety symptoms were observed to be correlated with the global efficiency of mid-distance subnetworks (edge 40–80 mm). Among patients with SCZ, integrated global network efficiency values were significantly correlated with depression and anxiety symptoms.
Orliac et al., 2013^52^	26 SCZ	DSM-IV	20/26 (76.9)	35.6 (8.9)	6	PANSS-general subscale (G6 item)	Intra-network connectivity strength of the salience network and default mode	Functional connectivity of the left striatum cluster showed a trend toward a negative correlation with PANSS-General Psychopathology subscale score. The same analysis with PANSS-General Psychopathology items revealed that the negative correlation was found only significant with the G6 item “depression”.
*task-fMRI*								
Athanassiou et al., 2021^b^ ^51^	62 SCZ SB– 42 SB + 20	DSM-5	SB– 36/42 (85.7) SB + 18/20 (90.0)	SB– N/A SB + N/A	6	PANSS- depression and anxiety subscale	Whole-brain functional activity	No significant correlation was observed between brain activations of the left median cingulate gyrus, left middle frontal gyrus, and left precuneus during an emotional processing task and affective symptoms.
Kvarta et al., 2021^42^	18 SAD & SSDs not specified	DSM-IV	10/18 (55.6)	40.8 (13.7)	5	MTSD	Whole-brain functional activity	Anticipatory stress by ankle-shock task-induced ventral anterior cingulate cortex cluster activation significantly and inversely correlated with trait depression scores in SSD.
Kirschner et al.,2016^43^	27 SCZ	DSM-IV	18/27 (66.7)	31.9 (7.1)	5	CDSS	Functional activity of the ventral striatum	When looking at potential confounding variables, a significant positive bivariate correlation between CDSS total score and left ventral striatum activation during reward anticipation while performing a Monetary Incentive Delay Task was found in individuals with SCZ.
Kumari et al., 2016^c ^^44^	63 SCZ & SAD Min Dep 25 Mild Dep 17 Sev Dep 21	DSM-IV	Min Dep 20/25 (80.0) Mild Dep 12/17 (70.6) Sev Dep 14/21 (66.7)	Min Dep 39.4 (9.8) Mild Dep 34.8 (8.2) Sev Dep 39.6 (10.6)	5	BDI-II	Whole-brain functional activity	BDI-II scores correlated significantly positively with activity during fearful expressions (fearful > no face) in the left thalamus extending to the para post-pre-central gyrus, putamen-globus pallidus, supramarginal gyrus, insula, and inferior-middle frontal gyrus. BDI-II scores also correlated significantly positively with activity for fearful expressions when compared with neutral (fearful > neutral) expressions in the right superior frontal gyrus extending to the middle/medial frontal, left precentral, and left anterior cingulate gyri. There was significantly higher activity across the thalamic and superior frontal gyrus clusters in the moderate-to-severe depression subgroup, relative to no/minimal, and mild depression.
Lindner et al., 2016^50^	36 SCZ	DSM-IV	23/36 (63.9)	30.6 (8.0)	7	BDI	Whole-brain functional activity	In a multiple regression analysis predicting the mean activation of right and left amygdala clusters in response to threat-related facial expression by the Scale for assessment of negative symptoms (SANS) global flat affect score, BDI, State-Trait-Anxiety-Inventory trait score, duration of illness, verbal intelligence, age and gender, SANS global flat affect score was the only significant predictor.
Stephan-Otto et al., 2016^45^	20 SCZ	DSM-IV	12/20 (60.0)	39.9 (11.5)	5	CDSS	Functional activity of vision-related brain regions	Activation in the right middle occipital gyrus during object perception was positively associated with CDSS and HAMRS. CDSS total score was found to be associated with increased activation in the bilateral calcarine gyrus, right precuneus, and left supramarginal gyrus during object perception, controlling for the effects of HAMRS.
Arrondo et al., 2015^46^	22 SCZ	DSM-IV	19/22 (86.4)	32.7 (7.6)	5	BDI	Whole-brain functional activity	A negative correlation between the severity of depression (BDI score) and ventral striatum activity during reward anticipation was found in the SCZ group.
Lee et al., 2015^d ^^47^	28 SCZ SH– 14 SH + 14	DSM-IV	SH– 11/14 (78.6) SH + 12/14 (85.7)	SH– 38.9 (7.3) SH + 43.6 (11.3)	4	CDSS	Whole-brain functional activity	In the self-harm patient group, activity in the left posterior cingulate cortex was negatively correlated with CDSS scores. This association was not found to be significant in the non-self-harm patient group.
Simon et al.,2010^48^	15 SCZ & SAD	DSM-IV	10/15 (66.7)	26.3 (5.4)	5	CDSS	Functional activity of medial orbitofrontal cortex and ventral striatum	A significant negative correlation was found between ventral striatum activation in patients with schizophrenia during the receipt of a reward while performing a monetary incentive delay task and CDSS.
*sMRI & rs-fMRI*								
Liang et al., 2023^e ^^49^	312 SCZ & SAD SCZ 178 SAD 134	DSM-IV	SCZ 124/178 (69.7) SAD 58 /134 (37.7)	SCZ 34.5 (12.0) SAD 36.3 (12.3)	5	MADRS	Whole-brain fractional amplitude of low-frequency fluctuations (fALFF)	In SCZ, MADRS scores were positively associated with fALFF in the thalamus and hippocampus. In SAD, MADRS scores were positively associated with fALFF in the lingual and frontal gyrus.

^†^the maximum QA score was 8 points, with lower scores reflecting a greater risk of bias

^a^study split SSDs sample by BPRS-18 total scores into groups of good outcome (18-31), moderate outcome (32-41), and poor outcome (≥42).

^b^study split SSDs sample by Columbia Suicide Severity Rating Scale; the presence of past suicidal behavior (SB+) vs. no past suicidal behavior (SB-).

^c^study split SSDs sample by BDI-II cutoff: no/minimal depression 0–13; mild depression score 14–19; moderate-to-severe depression score 20 or above.

^d^study split SSDs sample into self-harm (SH+) or no self-harm (SH-) group, based on any past self-initiated acts.

^e^study is multimodal; shown across both tables to demonstrate sMRI and rs-fMRI (bolded) results separately. Results reported for schizophrenia and schizoaffective disorder separately. gray rows indicate studies with null findings.

*SAD* Schizoaffective disorder, *SCZ* Schizophrenia, *BDI/BDI-II* Beck’s Depression Inventory, *BPRS* Brief Psychiatric Rating Scale, *CDSS* Calgary Depression Scale for Schizophrenia, *MADRS*

Montgomery-Asberg Depression Rating Scale, *MTSD* Maryland Trait and State Depression Scale, *PANSS* Positive and Negative Symptom Scale.

### Quality assessment

One study received a low score (<4 points)^34^, 15 studies received a moderate score (4–5 points)^35–49^, and 17 studies received a high score (6+ points)^27,50–65^ on the modified NOS. Validation of diagnoses by independent sources was frequently unreported, and additional points were lost relatively uniformly across the other evaluation criteria.

### Structural studies

**Fig. 3:**
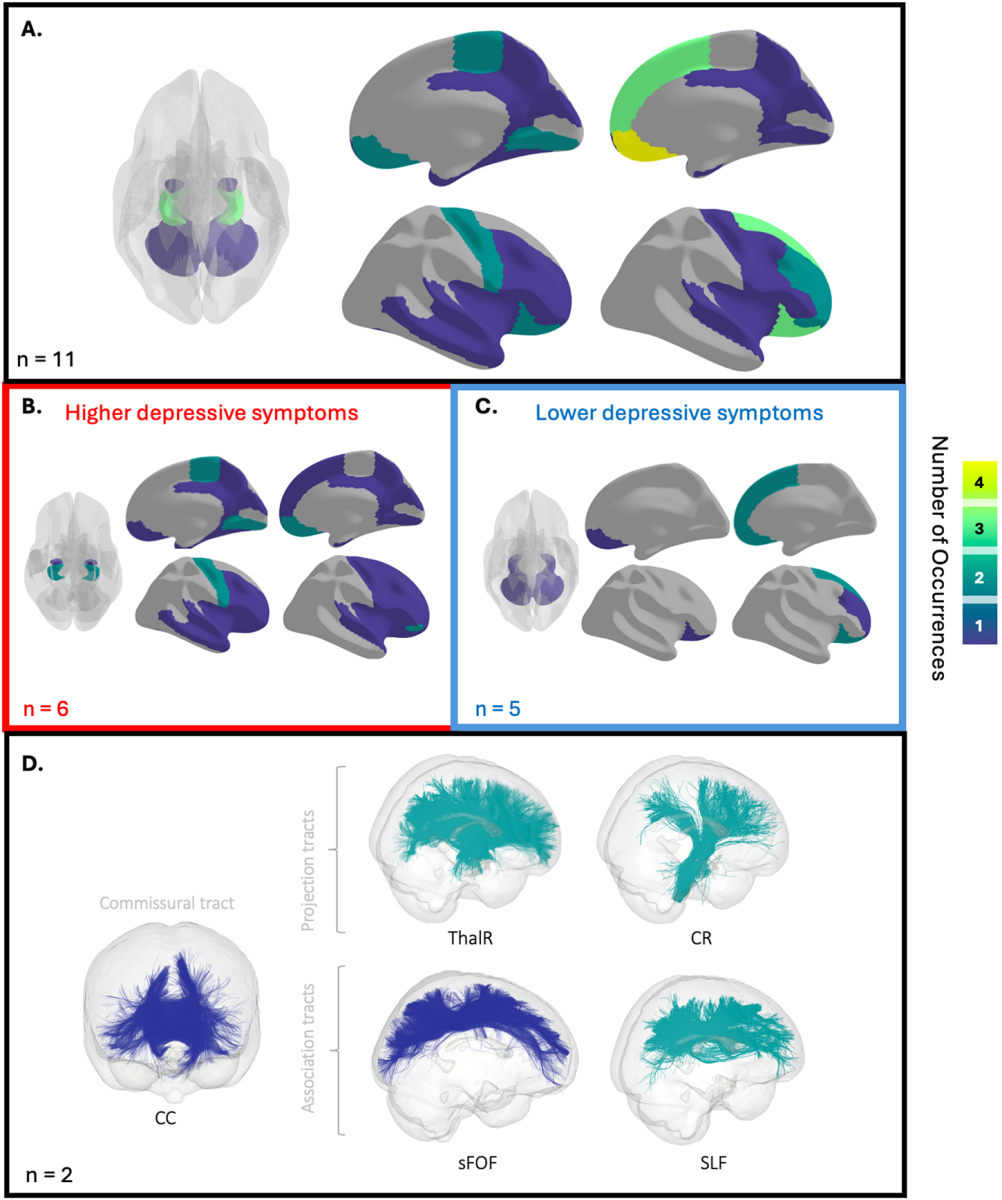
Summary of brain regions and white matter tracts most frequently associated with depressive symptoms in SSDs, based on 11 T1-weighted sMRI and 4 dMRI studies. The color scale corresponds to the frequency of the region or tract reported. **A** A schematic illustration of regions implicated in depressive symptoms in SSDs. Subcortical regions are shown through a glass brain, and cortical regions are displayed on the cerebral cortex, as per the Desikan-Killiany Cortical Atlas parcellation. Top regions include the bilateral hippocampus, as well as the right frontal areas. Implicated regions are further subdivided into positive (**B**) and negative (**C**) associations with depressive symptoms. **D** A schematic illustration of tracts implicated in depressive symptoms in SSDs. Tracts are shown overlaid on a glass brain, as per the O’Donnell Research Group Fiber Clustering White Matter Atlas parcellation. CC Corpus Callosum, CR Corona Radiata, sFOF Superior Fronto-Occipital Fasciculus, SLF Superior Longitudinal Fasciculus and ThalR Thalamic Radiation. Top tracts include CR, ThalR, and SLF. Note that association and projection tracts are displayed in the left hemisphere, and only the genus of the CC is shown for clarity. Both dMRI studies found positive correlations between white matter tract integrity and depressive symptoms.

Table 1 provides an overview of the included structural studies, encompassing key details such as the main clinical measure of depressive symptoms, the neuroimaging metric used, and a summary of the study’s findings. Figure 3 summarizes frequently implicated brain regions and white matter tracts.

#### Depressive symptom measures

The association between structural neuroimaging metrics with depressive symptoms was assessed using scales such as the CDSS (*n* = 4)^27,35,39,64^, the depression-anxiety subscale or depressive factor score of the Positive and Negative Syndrome Scale; PANSS (*n* = 3)^36,38,63^, the depression-anxiety subscale or affect factor score of the Brief Psychiatric Rating Scale; BPRS (*n* = 3)^37,59,62^, the HAMD (*n* = 3)^60,61,65^, and the Maryland Trait and State Depression Scale; MTSD (*n* = 1)^58^.

#### sMRI studies

Of the 14 structural studies, 10 employed metrics derived from T1-weighted sMRI such as morphology measurements related to volume, surface area, thickness, and size^27,35–37,60–65^.

Six of these studies associated depressive symptoms with brain morphology using a regional-specific approach^27,36,37,61–63^. Regions of interest (ROIs) included areas within the prefrontal cortex (namely, the dorsolateral prefrontal cortex (DLPFC) and orbitofrontal cortex)^27,61^, the hippocampus^36,63^, the amygdala^62^, and the cerebellum^37^. A negative correlation emerged between the severity of depressive symptoms and both volume^61^ and thickness^27^ within the prefrontal cortex. In the hippocampus, Bossù et al. found a negative association between the severity of depressive symptoms and volume^63^, while Smith et al. reported a positive correlation between depression scores and fissure size; a measure suggestive of abnormal neurodevelopment^36^. In the remaining studies, depression scores were positively associated with amygdalar volume^62^, and negatively associated with cerebellar volume^37^.

Four sMRI studies investigated the relationship between depressive symptoms and brain morphology using a whole-brain approach^35,60,64,65^. Kohler et al. reported increased left temporal lobe volume in patients with high depressive symptoms compared to those with low depressive symptoms^60^, whereas an association of lower volume in the superior frontal and orbitofrontal gyrus with higher depression scores was identified by Siddi et al.^64^. In a multivariate brain-behavior analysis, Buck et al. found specific patterns in females with SSDs, where fewer depressive symptoms were associated with changes in hippocampal subfields and varying thickness in specific cortical regions; such as lower thickness in the right superior temporal gyrus, entorhinal cortex, pars orbitalis, medial orbitofrontal gyrus and cingulate cortex, and high thickness in the left precentral gyrus, paracentral gyrus, cuneus, and lingual gyrus^35^. Notably, this brain-behavior pattern also correlated with fewer negative symptoms, though to a lesser extent. Finally, Wei et al. found that individuals with comorbid depressive symptoms had significantly greater gray matter volume in the left isthmus cingulate and posterior cingulate cortex, as well as increased surface area in the left isthmus cingulate, left superior parietal gyrus, and right cuneus compared to those without depressive symptoms^65^.

#### dMRI studies

Four studies used dMRI to assess the relationships between white matter tract integrity measures (i.e., FA, MD, radial diffusivity (RD), or white matter connectivity) and depressive symptoms in SSDs. Analytical methods across studies were highly heterogeneous. Chiappelli et al. used voxel-wise tract-based spatial statistics (TBSS)^58^, Amodio et al. used probabilistic tractography^38^, Long et al. used both voxel-wise TBSS and ROI probabilistic tractography^39^ and Joo et al. used whole-brain tractography^59^. Two of the four studies that used tractography did not find significant associations between alterations in white matter integrity and depressive symptoms in SSDs^38,59^. However, Chiappelli et al. found that greater experience of depression, termed ‘trait depression’, was positively linked to both the overall average FA values throughout the brain and FA values specific to four white matter pathways: the corona radiata, thalamic radiation, superior longitudinal fasciculus, and superior frontal-occipital tract^58^. Similarly, Long et al. found that patients with suicidal ideation exhibited elevated FA in several white matter tracts, including the corpus callosum, left anterior corona radiata, left superior corona radiata, and bilateral posterior corona radiata, as well as decreased MD in the splenium of the corpus callosum, bilateral posterior corona radiata, left posterior thalamic radiation and left superior longitudinal fasciculus^39^. However, this finding should be interpreted with caution, as suicidal ideation in psychosis could have multiple etiologies (i.e., delusion content, auditory verbal hallucination) despite being measured using the CDSS.

### Functional studies

**Fig. 4:**
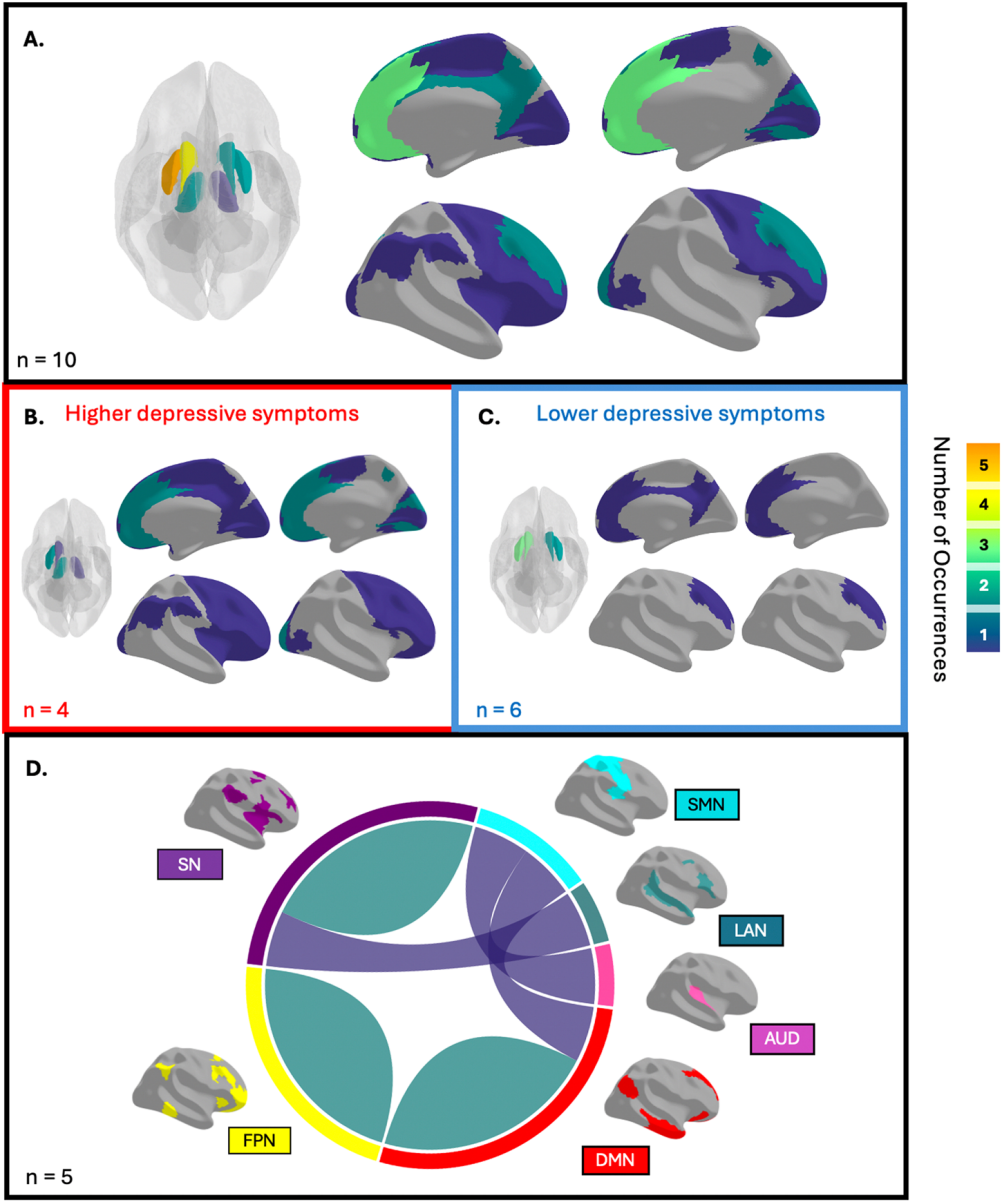
Summary of brain networks and regions most frequently associated with depressive symptoms in SSDs, based on 19 studies with fMRI. The color scale corresponds to the frequency of the region or network reported. **A** A schematic illustration of regions implicated in depressive symptoms in SSDs. Subcortical regions are shown through a glass brain, and cortical regions are displayed on the cerebral cortex, as per the Surface-Based Multimodal parcellation. Top regions include the left caudate and putamen and bilateral frontal area. Note that some studies investigated specific regions of interest (ROIs), and did not use a whole-brain approach. Implicated regions are further subdivided into positive (**B**) and negative (**C**) associations with depressive symptoms. **D** A schematic illustration of networks implicated in depressive symptoms in SSDs. Networks are displayed on the cerebral cortex, as per the Cole-Anticevic Brain-wide Network Partition, AUD Auditory Network, DMN Default Mode Network, FPN Frontoparietal Network, LAN Language Network, SMN Somatomotor Network, SN Salience Network. Networks were reported bilaterally but are displayed on the left hemisphere for clarity. Top network connections include within- DMN, FPN, and SN. All studies found negative correlations between network-based functional connectivity and depressive symptoms. One study identified both negative and positive associations with depressive symptoms (positive association found within-FPN, and between DMN-SMN).

Table 2 provides an overview of the included functional studies, encompassing key details such as the main clinical measure of depressive symptoms, the neuroimaging metric used, and a summary of the study’s findings. Figure 4 summarizes frequently implicated brain regions and networks, while Supplementary Fig. S5 provides a breakdown based on whether the findings are from resting-state or task-based analyses.

#### Depressive symptom measures

The association between functional neuroimaging metrics with depressive symptoms was assessed using scales such as the depression-anxiety subscale or depressive factor score of PANSS (*n* = 6)^34,41,51–53,57^, the CDSS (*n* = 5)^40,43,45,47,48^, the depression-anxiety subscale or affect factor of BPRS (*n* = 3)^54–56^, the Beck’s Depression Inventory; BDI/BDI-II (*n* = 3)^44,46,50^, the MTSD (*n* = 1)^42^.

#### rs-fMRI studies

Nine fMRI studies utilized metrics derived from resting-state fMRI (rs-fMRI) data, such as functional connectivity^34,40,52–56^, amplitude of low-frequency fluctuations (ALFF)^57^, and global/network efficiency^41^.

Five of these studies investigated associations of depressive symptoms with brain function using a specific seed- or a-priori network-based approach. In a lower-quality ROI-based analysis of resting state functional connectivity, Xu et al. found no significant correlation between depressive symptoms and the substantia nigra/ventral tegmental area^34^. However, in analyses of resting state functional connectivity based on specific networks of interest, depressive symptoms were linked to the default mode network (DMN)^55^, salience network^40,52^, and frontoparietal network (FPN)^53^ (often synonymous with the central executive network; CEN).

The remaining four studies used a whole-brain regional or network-level approach^41,54,56,57^. Analytical methods and findings across studies were variable. Li et al. demonstrated that an increase in ALFF, which quantifies the strength of low-frequency brain activity fluctuations, in the dorsolateral region of the superior frontal gyrus was significantly linked to a greater reduction in depression scores^57^. Doucet et al. showed a robust pattern of functional network connectivity strongly correlated with improvements in depressive symptoms, with higher within-DMN connectivity being a significant positive predictor, while reduced within-CEN and diminished connectivity between DMN and sensorimotor networks acted as important negative predictors^56^. Notably, this connectivity pattern also correlated with improvements in positive symptoms. Moreover, Lee et al. found the variance in depressive symptom severity can be explained by within-network connectivity of the salience network and connectivity between salience-language networks and somatomotor-auditory networks^54^. Lastly, Su et al. used graph theoretical analysis of networks to show depression symptoms were significantly correlated with the overall efficiency of brain network information processing^41^.

#### task-fMRI studies

Nine studies employed task-based fMRI to evaluate the relationship between functional brain activity and depressive symptoms in SSDs^42–48,50,51^; three of which investigated specific ROIs. Significant positive associations were found between functional activity in the ventral striatum during a monetary incentive delay task measuring reward processing^43,48^, and visual-related regions during an object perception task^45^ with depressive symptoms.

The remaining six studies used a whole-brain approach^42,44,46,47,50,51^. Two of the studies did not find any significant associations between brain activation and depressive symptoms^50,51^. Conversely, Lee et al. found that activity in the left posterior cingulate cortex was inversely correlated with overall depression scores^47^. Arrondo et al. demonstrated a negative correlation between depression severity and ventral striatum activity during reward anticipation^46^. Kumari et al. highlighted significant positive correlations between depression scores and brain activity in several regions while processing fearful expressions, including the left thalamus, para post-pre-central gyrus, putamen-globus pallidus, supramarginal gyrus, insula, inferior-middle frontal gyrus, and right superior frontal gyrus, extending to other frontal and cingulate gyri^44^. Moreover, higher activity was noted in thalamic and superior frontal gyrus clusters among patients with moderate-to-severe depression compared to those with milder levels of depression. Lastly, Kvarta et al. found a significant inverse correlation between anticipatory threat-induced ventral anterior cingulate cortex cluster activation and trait depression^42^.

### Multimodal study

A study with the largest sample size (*n* = 312) by Liang et al., employed a multimodal approach investigating both whole-brain fractional ALFF (fALFF) and gray matter volume in relation to depressive symptoms, assessed using the Montgomery-Asberg Depression Rating Scale (MADRS) (42). The authors investigated associations in schizophrenia and schizoaffective disorder groups separately and identified distinctions. In schizophrenia, elevated depression scores were linked to increased fALFF in the thalamus and hippocampus, as well as heightened gray matter volume in the insula and inferior frontal cortex. In schizoaffective disorder, higher depression scores were associated with increased fALFF and greater gray matter volume in the lingual and frontal gyrus.

## Discussion

We conducted a systematic review of 33 studies, comprising 14 structural MRI studies (10 sMRI and four dMRI), 18 fMRI studies, and one study analyzing both sMRI and fMRI, aiming to provide a comprehensive summary of the current neuroimaging research regarding depressive symptoms in individuals with SSDs. Our review underscored a notable gap in the literature, revealing a substantial lack of studies investigating the neurobiology of depression in SSDs. The relatively few studies that did explore imaging metrics of depressive symptoms demonstrated high variability and limited consistency across implicated neural correlates. These studies employed a diverse range of scales or assessments to measure depressive symptoms, an array of imaging modalities, and variable approaches to imaging analysis and statistical methods, posing a challenge for interpretation. For instance, regions that appeared more prominently in task-based fMRI studies versus resting-state (e.g., striatum) likely reflect the influence of emotional stimuli, activating areas involved in processing affective information, thus introducing potential biases. Nevertheless, findings delineated subcortical regions, specifically the striatum, thalamus, and hippocampus, as well as frontal regions as potentially implicated in the manifestation of depressive symptoms in SSDs. Notably, many of these correlates showed contrasting associations with depression across studies, which may be attributed to studies focusing on larger-scale brain areas, potentially overlooking nuances of sub-regions.

The subcortical and frontal areas highlighted in this review align with the results of several fMRI/MRI studies of MDD, consistently noting atypical morphology and functioning of such regions^66^. It has been suggested that these regions may be acting as central brain “hubs”, where impairments could lead to key symptomatology as observed in MDD (see Zhang et al. 2018, for an in-depth review)^66^. The involvement of the cortico-striatal-thalamo-cortical circuit in both SSDs^67,68^ and severe mood disorders, including MDD^69^, supports the notion that common abnormalities in these regions could reflect an overlapping feature of mood-related symptoms across diagnoses^70^. However, confidence in this shared neurobiology is constrained by methodological limitations in the literature.

A major challenge arises from the variation in scales and inclusion criteria used across studies to either identify patients with depressive symptoms or report on the severity of depression symptoms in SSDs. The studies identified in this review used a variety of tools to assess depressive symptoms in SSDs, with some originally developed for MDD and others for SSDs. A few studies classified patients as having depression with symptoms above a specified symptom scale cut-off, while no studies included or reported information regarding comorbid depressive disorders. When reporting depression severity, all studies reported an average total depression score and did not take into account specific symptoms that might be more relevant to SSDs. Prior work has suggested depressive symptoms in SSDs can be broken down into two dimensions: depression-hopelessness and pathological guilt^71^ which may have distinctive neural circuitry and treatment outcomes (Gallucci et al., accepted). Differentiation of these factors could unveil more consistent and clinically meaningful distinctions, emphasizing the importance of future research considering these separate dimensions rather than focusing on total depression scores. This approach could also provide insight into more general depressive symptoms in individuals with SSDs who might not fulfill the criteria for depressive disorders like MDD.

Another knowledge gap lies in understanding the extent to which negative symptoms may contribute to the overall picture of depressive symptoms. Many of the brain regions associated with depressive symptoms in our review have also been implicated in negative symptoms of SSD, such as structural brain abnormalities in the frontal and subcortical areas, along with functional alterations concentrated in the thalamocortical circuits^72^. Lako et al. have argued that depression scales designed for MDD may not effectively distinguish depressive symptoms from negative symptoms in SSDs, limiting our ability to adequately characterize these clinical phenotypes^73^. This conceptual overlap may contribute to heterogeneity seen across studies’ findings^11,74^. Our recent work, employing an advanced multivariate correlation approach, demonstrated distinct neural circuitry underlying depressive and negative symptoms (Gallucci et al., accepted). This provides evidence that these symptoms are separable constructs with differing neurobiological underpinnings. Further, brain stimulation treatments, such as rTMS targeting the DLPFC, have yielded substantial effects in mitigating depressive symptoms in MDD^26^, and preliminary support in SSDs^27^. This intervention has also demonstrated promising yet inconsistent efficacy in ameliorating negative symptoms in SSDs, suggesting potentially shared yet distinct pathophysiological mechanisms^75,76^.

Further limitations in the literature which significantly impacted interpretability should be acknowledged. There were considerable variations in the samples being studied, such as age, sex ratio, medication usage, and stage of illness (i.e., chronic versus first-episode). Greater attention should be devoted to examining subgroups within SSDs, such as first-episode patients or those who are drug-naive. A critical constraint across nearly all studies was the insufficient consideration for potential confounding factors, i.e., negative symptoms, as alluded to above. Notably, a large portion of relevant studies (23 out of 34) investigated depressive symptoms as secondary exploratory or post hoc analyses, lacking an SSD sample prospectively enriched with individuals experiencing depressive symptoms. This emphasizes the pressing need for more direct studies prioritizing the recruitment of such individuals, enabling a more nuanced examination of the underlying neurobiology.

## Conclusion and future directions

In our systematic review, we identified 33 studies focusing on neuroimaging research related to depressive symptoms in individuals with SSDs. We noted considerable variability and a lack of consistency amongst neural correlates. This heterogeneity may derive from assessment scales that fail to adequately distinguish between subdimensions of depressive symptoms. Our findings also suggest potential shared neurobiological underpinnings among depressive symptoms in SSDs and MDD. Given the relative scarcity of neuroimaging studies on depressive symptoms in SSDs and their inconsistent results, there is a clear need for research focused on directly investigating the neurobiology of depression in SSDs. Future studies may benefit from considering a more fine-grained and disorder-specific assessment of depressive symptoms in SSDs, rather than ‘total depression’ summary scores. Lastly, in light of preliminary evidence suggesting some neurobiological overlap between depressive symptoms in SSDs and MDD, a potential future direction may be to examine both unique and shared neural correlates across the two disorders. Exploring the neurobiology of individuals with SSDs and comorbid MDD is a severely understudied yet valuable avenue of research. While prior studies on MDD have noted irregularities in white matter tracts^77^ and brain networks^78^, we lack sufficient evidence to comment on how these abnormalities may relate to depressive symptoms in SSDs. The limited availability of studies using dMRI metrics or examining brain function at the network level underscores the necessity for further investigation. A better understanding of the neural correlates linked to depressive symptoms in SSDs could have pronounced implications, informing innovative treatment strategies tailored to alleviating depression specifically within SSDs, in contrast to the conventional but ineffective methods that were developed for MDD^11–13^.

### Supplementary information

Supplemental contents
