# Supplementary material for: A systematic review of structural and functional magnetic resonance imaging studies on the neurobiology of depressive symptoms in schizophrenia spectrum disorders
Source: Schizophrenia (Heidelb). 2024 Jul 4;10(1):59. doi: 10.1038/s41537-024-00478-w (PMC11222445; doi:10.1038/s41537-024-00478-w)
Supplement: Supplementary file 1 — Supplemental contents [file 41537_2024_478_MOESM1_ESM.docx]

Supplemental Contents

Supplemental Figure List**……………………………………………………………………….**

Figure S1 Search concepts: MeSH terms adapted for PsycINFO.

Figure S2 Search concepts: MeSH terms adapted for EMBASE.

Figure S3 Search concepts: MeSH terms adapted for Cochrane Library.

Figure S4 Search concepts: MeSH terms adapted for Web of Science.

Figure S5 Summary of brain networks and regions most frequently associated

with depressive symptoms in SSDs, based on 19 studies with fMRI stratified by

task or rest-state.

Supplemental Table Lists**……………………………………………………………………….**

Table S1: Modified Newcastle-Ottawa Scale

**
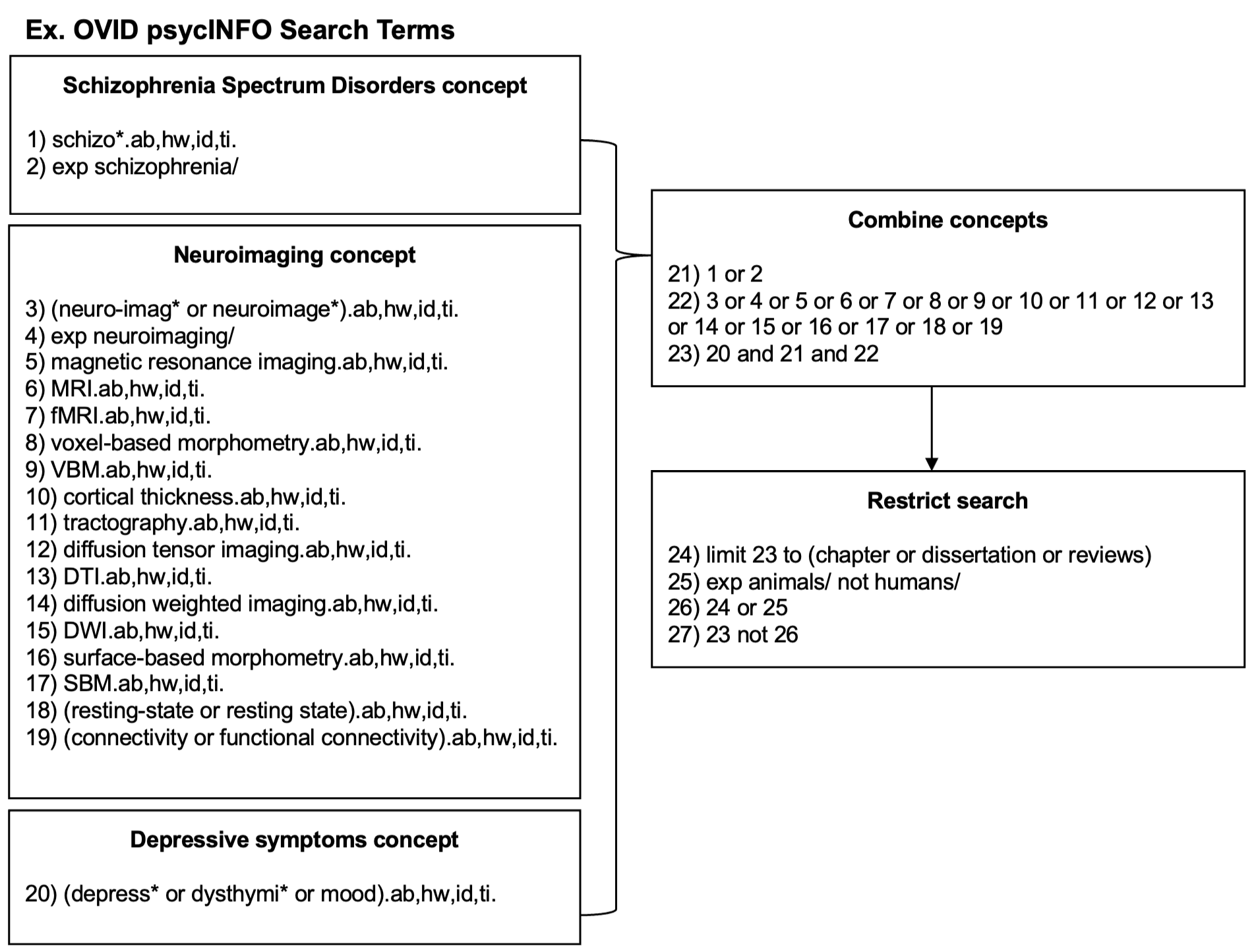
**

**Supplemental Figure S1. Search concepts: MeSH terms adapted for PsycINFO (Ovid)**. Ab indicates abstract; hw subject heading work; id, key concepts, ti, title.

**
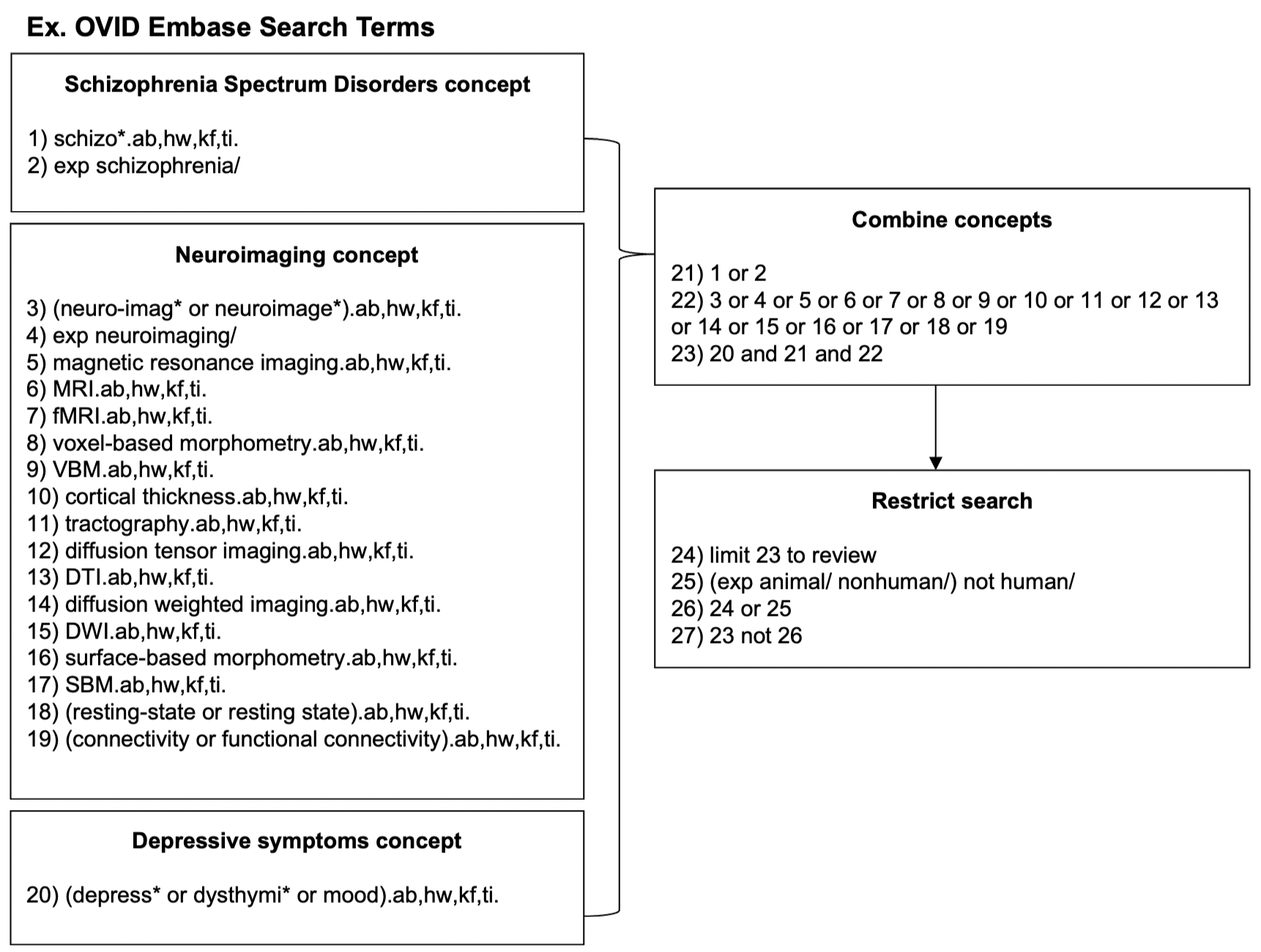
**

**Supplemental Figure S2. Search concepts: MeSH terms adapted for EMBASE (Ovid)**. Ab indicates abstract; hw subject heading work; kf, keyword heading word, ti, title.

**
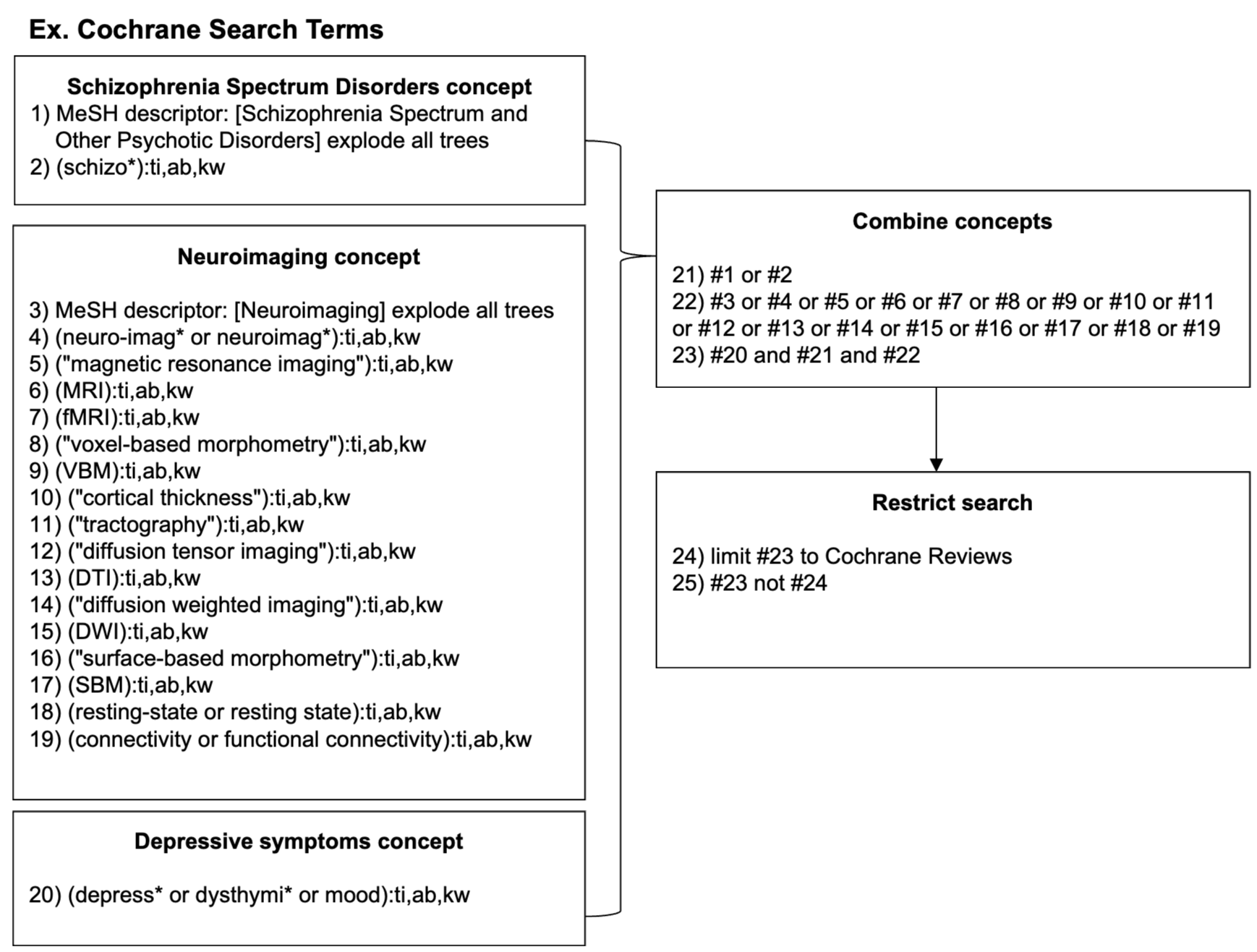
**

**Supplemental Figure S3. Search concepts: MeSH terms adapted for Cochrane Library**. Ab indicates abstract; kw keyword, ti, title.

**
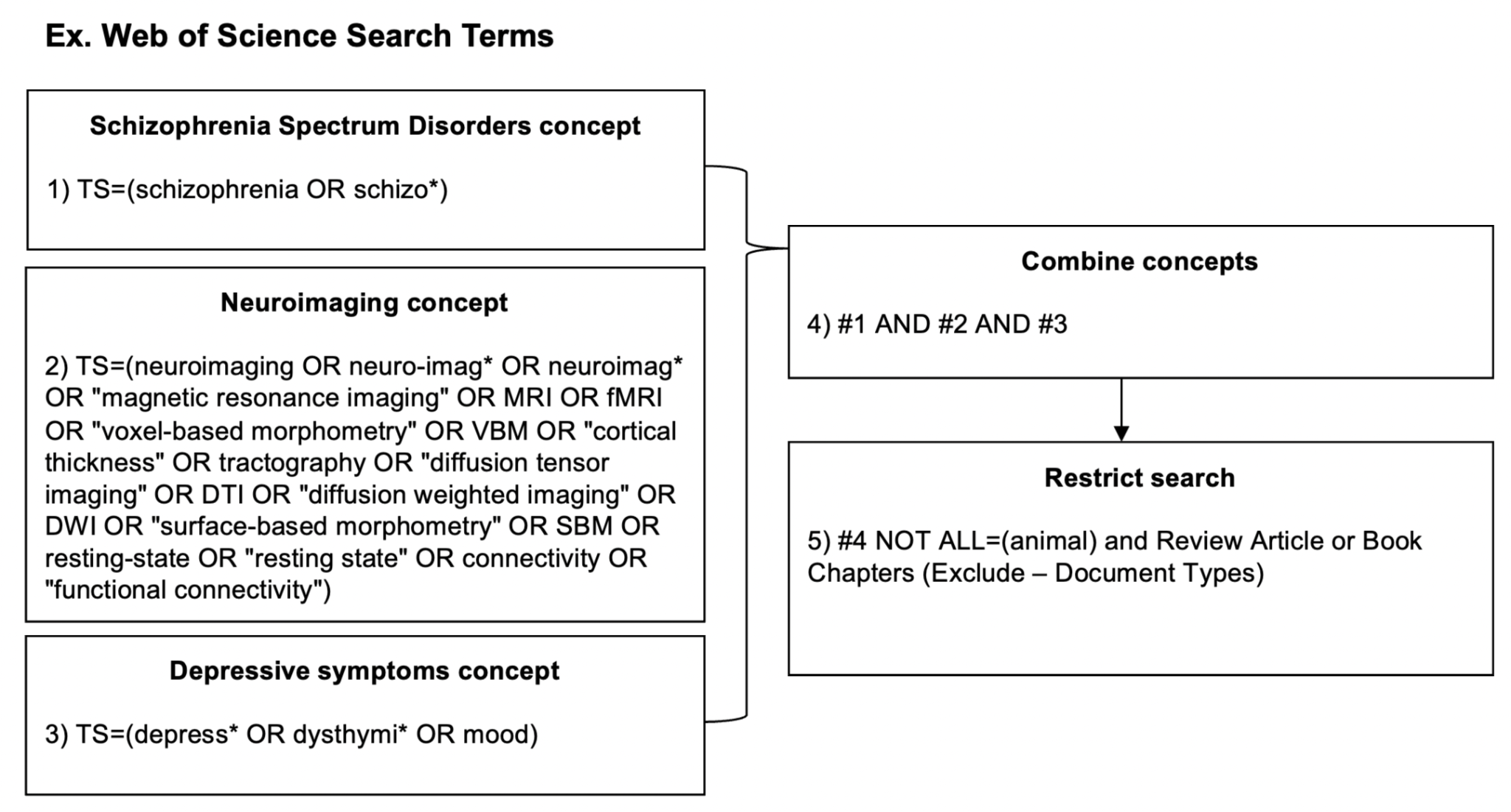
**

**Supplemental Figure S4. Search concepts: MeSH terms adapted for Web of Science** TS indicates searches title, abstract, author keywords, and keywords plus.


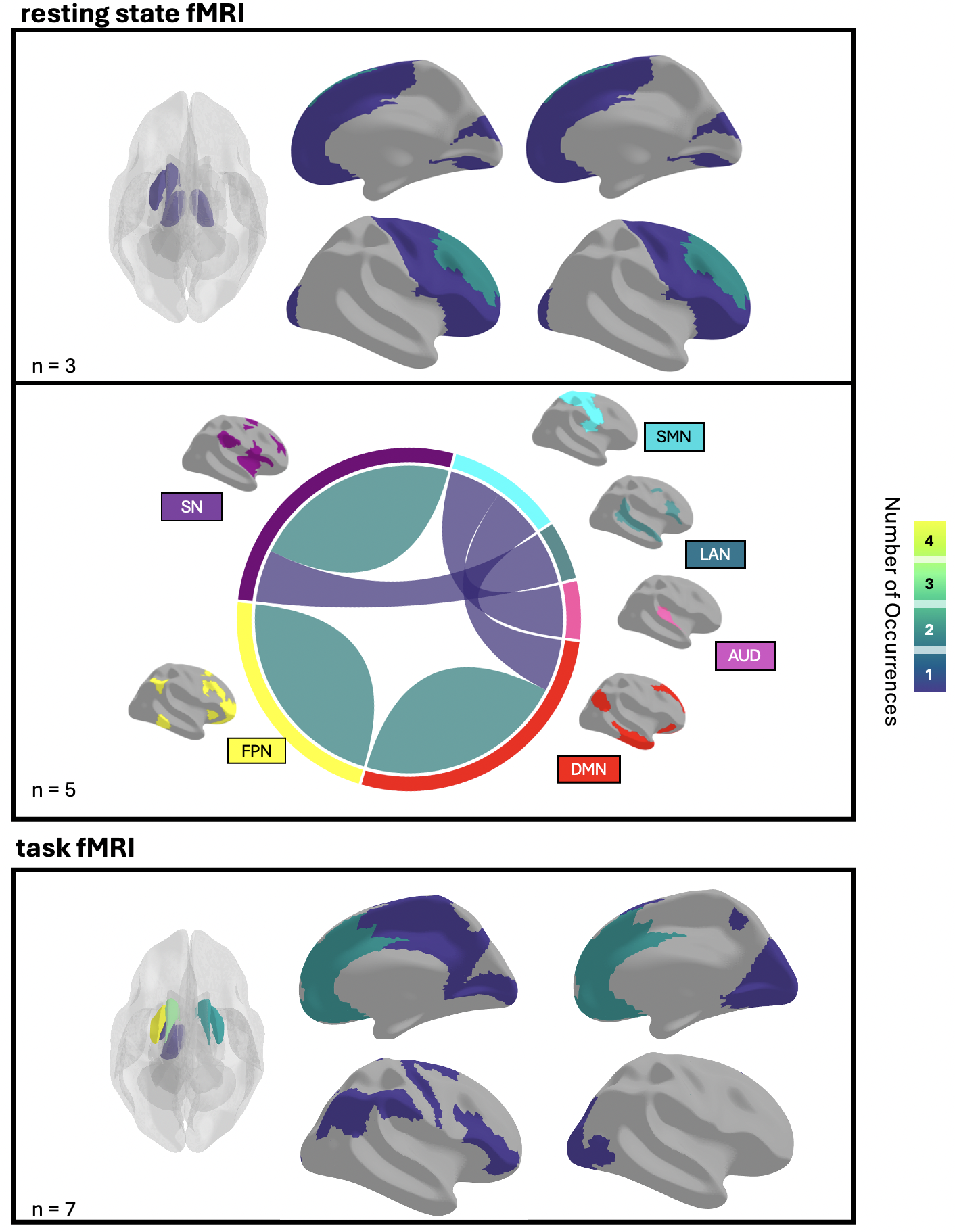


**Supplemental Figure S5. Summary of brain networks and regions most frequently associated with depressive symptoms in SSDs, based on 19 studies with fMRI stratified by task or resting-state.** The color scale corresponds to the frequency of the region or network reported. Subcortical regions are shown through a glass brain, and cortical regions are displayed on the cerebral cortex, as per the Surface-Based Multimodal parcellation. Networks are displayed on the cerebral cortex, as per the Cole-Anticevic Brain-wide Network Partition. Abbreviations: Auditory Network; AUD; Default Mode Network; DMN, Frontoparietal Network; FPN, Language Network; LAN, Somatomotor Network; SMN, Salience Network; SN. Networks were reported bilaterally but are displayed on the left hemisphere for clarity.

**Supplemental Table S1: Modified Newcastle-Ottawa Scale**

| **Score Description** | |
| --- | --- |
| 1  0 | ***Adequacy of the SSD definition*** Independent validation (1+ person or process) to ensure diagnostic accuracy using the DSM No description of independent validation process |
| 1  0 | ***Depth of SSD sample characterization***  Includes information such as neurological status, substance abuse, and comorbidities  No additional sample information provided |
| 1  0 | ***Representativeness of the SSD cohort***  Representative of the patient population  Select sample only (i.e., restricted to a specific sex/gender, race/ethnicity, etc.) |
| 1  0 | ***Inadequate sample size***  At least 30 SSD (if split SSD sample, each subgroup has n ≥ 30)  Less than 30 SSD |
| 1  0 | ***Medication summary***  Clear description of medication status  No description of medication status |
| 1  0 | ***Assessment of depressive symptoms***  Validated clinical scale or questionnaire  No description of depressive symptom assessment |
| 1  0 | ***Statistical test***  The statistical test used to analyze the data is clearly described and appropriate, and the measurement of the association is presented, including confidence intervals and/or the probability level (p-value)  The statistical test is not appropriate, not described, or incomplete |
| 1  0 | ***Correction for multiple comparisons***  Clear description of the process to correct for multiple comparisons in analyses  No description of the process to correct for multiple comparisons in analyses |
| **8** | *7+/8 good, 5+/8 moderate, 4-/8 poor* |
|  | |
